# Comparative evaluation of NeoPUTTY and mineral trioxide aggregate for full pulpotomy in mature permanent molars with irreversible pulpitis: a randomized clinical study

**DOI:** 10.2340/biid.v13.46677

**Published:** 2026-08-13

**Authors:** Shiwani Chawla, Ruchi Singhal, Ritu Namdev, Chanchal Kumari, Khusboo Chhanna

**Affiliations:** Department of Pediatric and Preventive Dentistry, Post Graduate Institute of Dental Sciences, Rohtak, Haryana, India

**Keywords:** Vital pulp therapy, pulpotomy, irreversible pulpitis, NeoPUTTY, mineral trioxide aggregate, mature permanent molars, pediatric dentistry, bioceramics

## Abstract

**Objective:**

To compare the clinical and radiographic outcomes of NeoPUTTY and mineral trioxide aggregate (MTA) as medicaments for full pulpotomy in mature permanent molars with irreversible pulpitis in children.

**Materials and Methods:**

Forty-six mature permanent molars from 46 children aged 9–14 years (one tooth per child) were randomly allocated to NeoPUTTY (*n* = 23) or MTA (*n* = 23) groups. Full pulpotomy was performed under rubber dam isolation followed by immediate composite restoration. Clinical and radiographic outcomes were evaluated at 1, 3, 6, and 12 months and at 6 and 12 months, respectively. Postoperative pain was assessed at 24 h using a visual analog scale (VAS). Hemostasis time was recorded intraoperatively. Per-protocol analysis was performed using an independent *t*-test, Mann–Whitney U test, Chi-square test, and Fisher’s exact test (*p* < 0.05). The trial was registered on ClinicalTrials.gov (NCT07702942).

**Results:**

Forty-four teeth (22 per group) completed the final analysis; one participant from each group was lost to follow-up. Mean VAS scores decreased significantly within 24 h in both groups (*p* < 0.001), from 6.48 ± 1.73 to 0.91 ± 1.12 (NeoPUTTY) and from 6.17 ± 1.47 to 0.65 ± 0.78 (MTA), with no significant intergroup difference. Overall success at 12 months was 95.5% in both groups (21/22; 95% confidence interval [CI]: 77.2–99.9; *p* = 1.000). Intra- and inter-examiner radiographic agreement was almost perfect (κ = 0.87 and 0.83). Treatment outcome was not significantly associated with jaw location, sex, pain duration, hemostasis time, caries depth, or Wolters pulpitis grade.

**Conclusion:**

Within the limitations of this study and 12-month follow-up, no statistically significant differences were detected between NeoPUTTY and MTA in postoperative pain reduction or clinical/radiographic success after full pulpotomy in mature permanent molars with irreversible pulpitis. Adequately powered non-inferiority or equivalence trials with longer follow-up are required to confirm these preliminary findings.

## Introduction

Dental caries is a highly prevalent biofilm-mediated disease in which bacterial metabolism of dietary sugars produces acids that demineralize tooth structure [1]. In deep carious lesions, bacterial diffusion through dentinal tubules toward the pulp induces a localized immune-inflammatory response, increasing intrapulpal pressure and pain, clinically manifested as pulpitis [2]. Traditionally, pulpitis has been classified as reversible or irreversible, with irreversible pulpitis in mature permanent teeth regarded as a non-healable condition and conventionally treated by root canal therapy (RCT) [3]. However, histopathologic studies demonstrate that pulpal inflammation is frequently spatially localized. Ricucci et al. showed that teeth clinically diagnosed with irreversible pulpitis may exhibit necrotic or bacterially colonized tissue limited to the coronal pulp, while healthy or minimally inflamed vital pulp persists apically [4].

This clinico-histological discrepancy challenges the reversible–irreversible model and supports preservation of vital radicular pulp after removal of inflamed coronal tissue. Based on these biological insights, Wolters et al. proposed a revised classification of pulpitis that describes the disease as a continuum of inflammatory stages – initial, mild, moderate, and severe – emphasizing the healing potential of the remaining vital pulp and guiding appropriate treatment selection [5]. Consistent with this concept, the American Association of Endodontists (AAE) and the European Society of Endodontology (ESE) recognize pulpotomy as a definitive biologic treatment option for mature permanent teeth with symptoms of irreversible pulpitis [6, 7].

The predictability of pulpotomy depends on achieving effective hemostasis, establishing an adequate coronal seal, and using bioactive calcium silicate–based materials [8]. Mineral trioxide aggregate (MTA) has long been regarded as the gold standard material because of its biocompatibility, sealing ability, and dentinogenic potential; however, its clinical use is limited by difficult handling, prolonged setting time, and tooth discoloration related to bismuth oxide [9–11]. These limitations have driven the development of premixed bioceramic materials designed to retain MTA’s biological properties while improving handling. NeoPUTTY® (NuSmile®, Houston, TX, USA) is a next-generation premixed calcium silicate bioceramic manufactured in an FDA-registered, ISO 13485-certified facility in accordance with applicable dental device quality standards. It consists of tricalcium and dicalcium silicates in a water-free vehicle with tantalum oxide radiopacifier. Its moisture-activated setting reaction provides extended working time, improved handling, and reduced chair-side time [12–14].

Available clinical evidence on NeoPUTTY in pediatric dentistry remains limited but encouraging. Randomized controlled trials in primary molars have reported pulpotomy success rates exceeding 90% [13, 15, 16] while indirect pulp treatment studies have reported success rates of 90–96% [17, 18]. Evidence in mature permanent teeth is more limited; one randomized clinical trial evaluating NeoPUTTY pulpotomy in mature permanent molars with spontaneous symptomatic pulpitis reported 12-month success rates of 98.9% for full pulpotomy and 84.5% for partial pulpotomy, with full pulpotomy demonstrating significantly superior outcomes [19]. Case reports have additionally documented its use in regenerative endodontic procedures, apexification, and root-end surgery [20–22].

Nevertheless, direct comparative clinical evidence between NeoPUTTY and the gold-standard MTA in mature permanent molars remains scarce. Therefore, the present randomized clinical trial was conducted to compare their clinical and radiographic outcomes as pulpotomy medicaments in mature permanent molars with irreversible pulpitis. The null hypothesis was that no significant difference would exist between the two materials in clinical and radiographic success at 12 months.

## Materials and methods

### Study design and ethical approval

This prospective, parallel-arm randomized clinical study was conducted with a 12-month follow-up period. The study was carried out in accordance with the principles of the Declaration of Helsinki and Good Clinical Practice guidelines. Ethical approval was obtained from the Institutional Ethics Committee of Post Graduate Institute of Dental Sciences, Rohtak (Approval No. PGIDS/BHRC/24/48). The study was retrospectively registered at the clinical trials website (http://www.clinicaltrials.gov) with the number NCT07702942. Written informed consent was obtained from the parents or legal guardians of all participating children prior to enrollment.

### Eligibility criteria

#### Inclusion criteria

Inclusion criteria comprised systemically healthy children aged 9–14 years. Eligible teeth were mature permanent maxillary or mandibular molars with complete root development and confirmed closed apices on a periapical radiograph, with sufficient residual coronal tooth structure for a definitive restoration. A clinical diagnosis of symptomatic irreversible pulpitis was established according to AAE diagnostic criteria, characterized by spontaneous pain, lingering thermal pain, or referred pain; pulpitis severity was graded as moderate or severe using the Wolters et al. classification based on character and duration of pain, response to cold testing (Endo-Frost, Coltène/Whaledent), analgesic requirement, and percussion sensitivity. Teeth presented with deep carious lesions extending into the inner third of dentine with risk of pulp exposure, or extremely deep carious lesions in which pulp exposure was unavoidable during operative treatment [23, 24]. Radiographically, eligible teeth showed a normal periapical appearance or mild periapical changes restricted to slight widening of the periodontal ligament space or subtle trabecular alterations, without loss of lamina dura continuity or frank periapical radiolucency, and were free of advanced periodontal disease. Parents or legal guardians of all participating children provided written informed consent prior to enrollment.

#### Exclusion criteria

Exclusion criteria included incomplete root formation or open apices, insufficient residual coronal structure for restoration, pathological tooth mobility, presence of a sinus tract or soft-tissue swelling, and radiographic evidence of established apical periodontitis characterized by frank periapical radiolucency, loss of lamina dura continuity, or extensive periapical trabecular bone destruction. Teeth with evidence of internal or external root resorption were excluded. Additional exclusion criteria were known hypersensitivity to any material used in the study, absence of parental or guardian informed consent, and failure to achieve hemostasis within 25 min of coronal pulp amputation.

### Sample size calculation

Sample size was calculated using OpenEpi software (Version 3.01) based on a reported pulpotomy success rate of 97.1% [13]. With a confidence level of 80% and a margin of error of 5%, a minimum of 19 teeth per group was required. To account for possible dropouts and loss to follow-up, a 20% attrition rate was added, resulting in a final sample size of 46 teeth (23 per group). Because only one tooth per child was included and the number of eligible cases fulfilling the strict inclusion criteria was limited, a formal superiority, non-inferiority, or equivalence sample size calculation was not feasible.

### Randomization and blinding

Eligible teeth were allocated to Group I (NeoPUTTY) or Group II (MTA) by simple randomization using the lottery method in a 1:1 ratio. Before recruitment, an independent person not involved in recruitment, treatment, or outcome assessment prepared equal numbers of identical slips labeled ‘NeoPUTTY’ and ‘MTA’, mixed them thoroughly, and drew them randomly to determine group allocation for each participant. Allocation concealment was ensured using sequentially numbered, opaque, sealed envelopes (SNOSE), opened only after enrollment and intraoperative confirmation of eligibility. Participants and parents were blinded to group allocation as both interventions followed an identical standardized clinical protocol and the allocated material was not disclosed. Operator blinding was not feasible due to differences in material handling. To minimize radiographic detection bias, radiographs were anonymized and evaluated in random order using predefined biological outcome criteria rather than material appearance. Although the materials may show slight differences in radiopacity, assessors were instructed not to infer group allocation from the pulpotomy material and to assess only periapical status, periodontal ligament changes, and internal or external resorption.

### Clinical procedure

**Figure 1:**
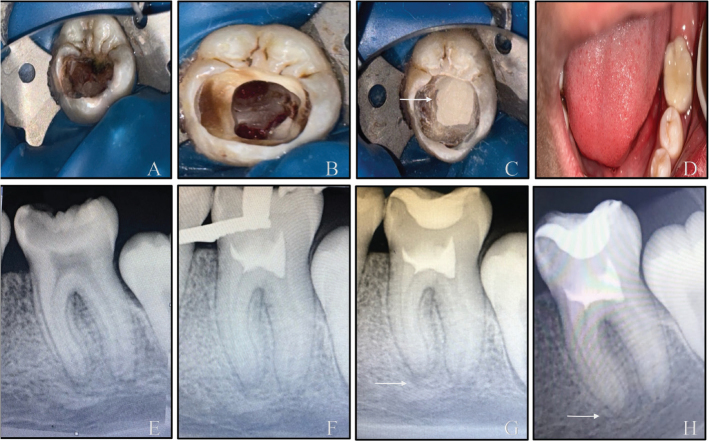
NeoPUTTY group. (A) Preoperative clinical photograph showing the carious lesion. (B) Haemostasis achieved using 5% NaOCl after full pulpotomy. (C) NeoPUTTY placement over the radicular pulp stumps (White arrow). (D) Postoperative composite restoration. (E) Preoperative periapical radiograph. (F) Immediate postoperative radiograph showing NeoPUTTY placement. (G) Six-month follow-up radiograph showing stable periapical status with no progressive radiographic pathology (White arrow). (H) Twelve-month follow-up radiograph showing restoration of normal periapical trabecular bone pattern (White arrow).

**Figure 2:**
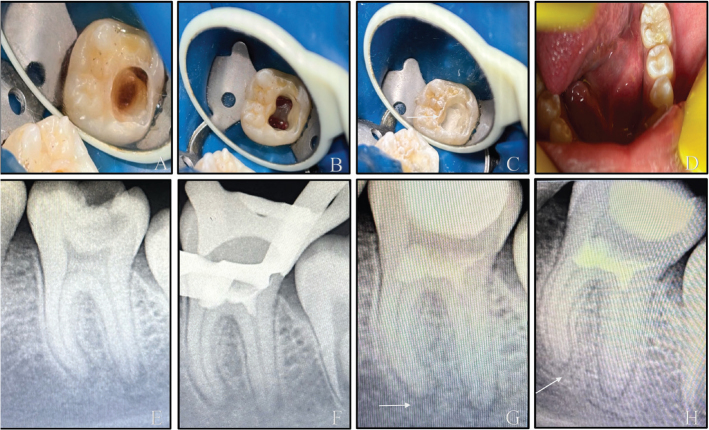
MTA group. (A) Preoperative clinical photograph showing the carious lesion. (B) Haemostasis achieved using 5% NaOCl after full pulpotomy. (C) MTA placement over the radicular pulp stumps (White arrow). (D) Postoperative composite restoration. (E) Preoperative periapical radiograph. (F) Immediate postoperative radiograph showing MTA placement. (G) Six-month follow-up radiograph showing improvement in subtle periapical trabecular changes (White arrow). (H) Twelve-month follow-up radiograph showing restoration of normal periapical trabecular bone pattern (White arrow).

Pre-operative pain (T_0_) was recorded using the Visual Analog Scale (VAS), a 10-point scale ranging from 0 (no pain) to 10 (worst imaginable pain) [25]. Wolters pulpitis grade was assigned prior to access cavity preparation based on a combination of clinical signs and symptoms – including character and duration of pain, response to cold testing, and presence or absence of percussion tenderness – in accordance with the criteria proposed by Wolters et al. [5]. Local anesthesia was administered with 2% lignocaine containing 1:80,000 adrenaline, followed by rubber dam isolation. Caries removal was performed with a high-speed sterile round carbide bur under water coolant, followed by access cavity preparation and pulp chamber deroofing. The coronal pulp tissue was amputated using a fresh slow-speed sterile round bur or a sharp sterile spoon excavator, until the canal orifices were clearly visualized and free of residual pulp tissue. Pulp vitality was verified by the presence of bright red bleeding, with active hemorrhage observed from all canals following complete coronal pulp amputation. Hemostasis was achieved using cotton pellets moistened with 5% sodium hypochlorite (NaOCl), a readily available concentration within the 0.5–5% range recommended by the ESE position statement for vital pulp therapy [26, 27]. Time taken to achieve hemostasis was recorded and categorized into three predefined intervals: ≤5, 5–10, and >10 min. The maximum permissible hemostasis time was set at 25 min; cases in which hemostasis could not be achieved within this period were planned for root canal treatment and excluded from the study**.** Following achievement of hemostasis, pulpotomy medicaments were placed according to random allocation. In group 1, a 2–3 mm thick layer (minimum 1.5 mm) of premixed NeoPUTTY (NuSmile®, USA) was placed over the pulpal floor using a plastic filling instrument and was adapted using a moist cotton pellet. In group 2, Mineral Trioxide Aggregate (MTA Angelus®, Brazil) was mixed according to the manufacturer’s instructions and placed to a thickness of 2–3 mm; a moist cotton pellet was placed over the MTA for 15 min to allow initial setting (Figures 1 and 2).

In both groups, a glass ionomer cement base was placed over the pulpotomy material, followed by definitive restoration with resin composite in the same visit to ensure an immediate and durable coronal seal.

### Follow-up and outcome assessment

Patients were assessed clinically immediately after treatment and at 1, 3, 6, and 12 months, with radiographic evaluation at 6 and 12 months. Clinical success was defined as the absence of pain, percussion tenderness, swelling, sinus tract, or pathological mobility. Postoperative pain (T1) was recorded using VAS at 24 h; at subsequent follow-up visits (1, 3, 6, and 12 months), pain was assessed descriptively as part of routine clinical examination – its presence at any visit constituting a criterion for clinical failure – but was not subjected to quantitative VAS scoring. Radiographic success was defined as stability or improvement in periapical bony architecture, including reduction or resolution of pre-existing periapical radiolucency and normalization of the periodontal ligament space, with no evidence of new or progressive periapical radiolucency or internal or external root resorption. Radiographs were independently evaluated by calibrated, blinded examiners, and intra- and inter-examiner reliability were assessed using Cohen’s kappa coefficient.

### Statistical analysis

A per-protocol analysis was performed, and participants lost to follow-up were excluded from the final outcome analysis. Data were entered into Microsoft Excel and analyzed using IBM SPSS Statistics software (version 31.0). Descriptive statistics were calculated for all variables, and normality of data was assessed using the Shapiro–Wilk test. For baseline comparison between the NeoPUTTY and MTA groups, normally distributed continuous variables (age) were analyzed using the independent samples *t*-test. Categorical baseline variables, including sex, jaw location, caries depth, Wolters’ grade of pulpitis severity, and duration of preoperative pain, were analyzed using the Chi-square test, while Fisher’s exact test was applied when expected cell frequencies were small. For intergroup outcome comparison, non-normally distributed variables including postoperative pain scores (VAS) and hemostasis time were analyzed using the Mann–Whitney U test. Intragroup reduction in pain intensity between the preoperative and 24-h postoperative time points (T0–T1) was evaluated using the paired t-test. Associations between these clinical variables and overall treatment success were analyzed using Fisher’s exact test. A *p*-value < 0.05 was considered statistically significant.

## Results

**Figure 3:**
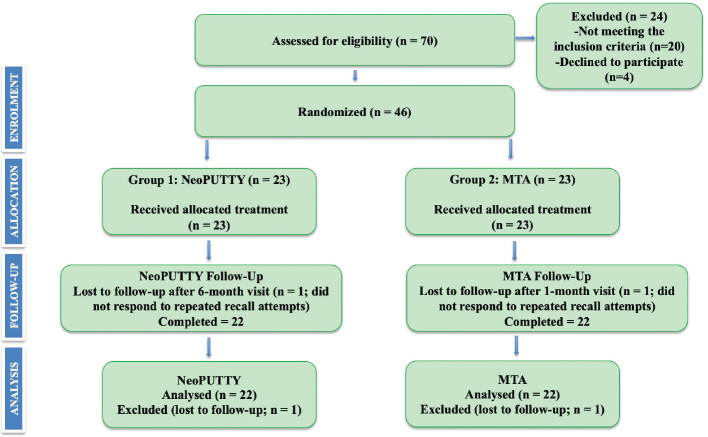
CONSORT flow diagram of participant enrollment, randomization, follow-up, and analysis.

Forty-six children aged 9–14 years with mature permanent molars diagnosed with irreversible pulpitis were randomized to the NeoPUTTY (*n* = 23) or MTA (*n* = 23) groups. One participant in the MTA group (14-year-old male) was lost to follow-up after the 1-month visit, and one participant in the NeoPUTTY group (9-year-old female) was lost to follow-up after the 6-month visit; both participants did not respond to repeated recall attempts. Forty-four teeth (22 per group) completed the 12-month follow-up and were included in the per-protocol analysis (Figure 3). Radiographic evaluation demonstrated excellent reliability, with intra-examiner (κ = 0.87) and inter-examiner agreement (κ = 0.83) indicating high consistency in radiographic assessment. No adverse events were reported in either group during the study period.

### Baseline characteristics

The groups were comparable at baseline. Mean age was 10.7 ± 1.48 years in the NeoPUTTY group and 11.5 ± 1.50 years in the MTA group (*p* = 0.100). The two groups were statistically comparable at baseline across all evaluated parameters, including age, gender distribution, jaw involvement, caries depth, Wolters’ pulpitis grade, duration of preoperative pain, and baseline pain intensity measured using the VAS, with no significant intergroup differences observed (*p* > 0.05) (Table 1).

**Table 1:** Baseline distribution of demographic and clinical variables.

Variable	Category	NeoPUTTY (*n* = 22)	MTA (*n* = 22)	*p*
**Age (years)**	Mean ± SD	10.7 ± 1.48	11.5 ± 1.50	0.100
**Sex**	Female	8	11	0.552
	Male	14	11	
**Jaw**	Mandibular	18	16	0.730
	Maxillary	4	6	
**Caries depth**	Deep	10	13	0.238
	Extremely deep	12	9	
**Wolters’ grade**	Moderate	11	12	0.768
	Severe	11	10	
**Duration of preoperative pain**	≤ 7 days	6	3	0.635
	8–14 days	9	10	
	≥ 15 days	7	9	
**Preoperative pain intensity (VAS)**	Mean ± SD	6.48 ± 1.73	6.17 ± 1.47	0.532

MTA: mineral trioxide aggregate; SD: Standard deviation; VAS: visual analogue scale.

Statistical significance at *p*-value < 0.05; final analysis based on 44 participants who completed the 12-month follow-up (NeoPUTTY *n* = 22; MTA *n* = 22).

### Intragroup clinical and radiographic changes

Intragroup analysis revealed a significant reduction in pain within 24 h in both groups (*p* < 0.001) (Table 2). Mean VAS scores decreased from 6.48 to 0.91 in the NeoPUTTY group and from 6.17 to 0.65 in the MTA group. Complete pain relief was achieved within 1–4 days in all patients except one. One clinical failure occurred in the NeoPUTTY group involving tooth #26 in a 12-year-old male presenting with severe Wolters’ grade pulpitis, deep caries, hemostasis time of 8 min, and preoperative VAS score of 9; the tooth presented with persistent spontaneous pain at the 1-month follow-up and was subsequently managed with root canal treatment. Radiographically, most teeth demonstrated stability or improvement in periapical bony architecture over the 12-month period. One radiographic failure occurred in the MTA group involving tooth #46 in a 12-year-old male presenting with severe Wolters’ grade pulpitis, deep caries, hemostasis time of 8.5 min, and preoperative VAS score of 6; the tooth remained clinically asymptomatic but demonstrated a new periapical radiolucency at the 12-month evaluation and was subsequently scheduled for root canal treatment.

**Table 2:** Intergroup comparison of clinical and radiographic outcomes.

Parameter	NeoPUTTY (*n* = 22)	MTA (*n* = 22)	*p*
**Postoperative pain (VAS) at 24 h (T1) Median (IQR) / Mean ± SD**	1 (0–2)/0.91 ± 1.12	1 (0–1)/0.65 ± 0.78	0.583
**Intragroup pain reduction (T0** **→** **T1)**	6.48 **→** 0.91	6.17 **→** 0.65	< 0.001 (both)
**Haemostasis time (min) Median / Mean ± SD**	8.00/8.15 ± 3.04	8.00/8.13 ± 2.24	0.774
**Clinical success at 12 months (95% CI)**	21/22 = 95.5% (77.2–99.9)	22/22 = 100% (84.6–100)	1.000
**Radiographic success at 12 months (95% CI)**	22/22 = 100% (84.6–100)	21/22 = 95.5% (77.2–99.9)	1.000
**Overall success at 12 months (95% CI)**	21/22 = 95.5% (77.2–99.9)	21/22 = 95.5% (77.2–99.9)	1.000

T0 = preoperative; T1 = 24-h postoperative.

MTA: mineral trioxide aggregate; VAS: Visual Analogue Scale; SD: standard deviation; IQR: interquartile range; CI: confidence interval.

Intergroup difference in overall success rate: 0% (95% CI: −12.3 to +12.3; Statistical significance at *p* < 0.05.

### Intergroup outcome comparison

Intergroup comparison demonstrated no statistically significant difference between NeoPUTTY and MTA with respect to intraoperative hemostasis time, postoperative pain reduction, and overall success rate at 12 months. Mean hemostasis time did not differ significantly (NeoPUTTY: 8.15 ± 3.04 min; MTA: 8.13 ± 2.24 min; *p* = 0.774). The maximum hemostasis time recorded across both groups was 15 min. No case exceeded the predefined protocol threshold of 25 min. Similarly, mean 24-h postoperative pain scores were comparable between groups (NeoPUTTY: 0.91 ± 1.12; MTA: 0.65 ± 0.78; *p* = 0.583). At 12 months, clinical success was 95.5% in the NeoPUTTY group and 100% in the MTA group, while radiographic success was identical in both groups (95.5%). Overall combined success at 12 months was identical in both groups (95.5%, 21/22; 95% confidence interval [CI]: 77.2–99.9), with one clinical failure in the NeoPUTTY group at 1 month and one radiographic failure in the MTA group at 12 months (*p* = 1.000) (Table 2).

### Association between clinical variables and treatment outcome

Analysis of clinical variables showed no significant association between treatment success and jaw location, sex, duration of preoperative pain, hemostasis time, caries depth, or Wolters’ pulpitis grade (*p* > 0.05 for all comparisons) (Table 3). Overall success rates remained high across all categories, ranging from 90.0 to 100%.

**Table 3:** Association between clinical variables and treatment success in NeoPUTTY and MTA groups.

Variable	Category	NeoPUTTY success *n/N* (%)	MTA success *n/N* (%)	Overall success *n/N* (%)
**Jaw**	Mandibular	18/18 (100)	15/16 (93.8)	33/34 (97.1)
	Maxillary	3/4 (75.0)	6/6 (100)	9/10 (90.0)
	*p*	0.182	1.000	0.407
**Sex**	Female	8/8 (100)	11/11 (100)	19/19 (100)
	Male	13/14 (92.9)	10/11 (90.9)	23/25 (92.0)
	*p*	1.000	1.000	0.498
**Duration of preoperative pain**	≤ 7 days	6/6 (100)	3/3 (100)	9/9 (100)
	8–14 days	8/9 (88.9)	10/10 (100)	18/19 (94.7)
	≥ 15 days	7/7 (100)	8/9 (88.9)	15/16 (93.8)
	*p*	1.000	0.545	1.000
**Haemostasis time**	≤ 5 min	5/5 (100)	3/3 (100)	8/8 (100)
	5–10 min	12/13 (92.3)	15/16 (93.8)	27/29 (93.1)
	> 10 min	4/4 (100)	3/3 (100)	7/7 (100)
	*p*	1.000	1.000	1.000
**Caries depth**	Deep	9/10 (90.0)	12/13 (92.3)	21/23 (91.3)
	Extremely deep	12/12 (100)	9/9 (100)	21/21 (100)
	*p*	0.455	1.000	0.489
**Wolters’ grade**	Moderate	11/11 (100)	12/12 (100)	23/23 (100)
	Severe	10/11 (90.9)	9/10 (90.0)	19/21 (90.5)
	*p*	1.000	0.455	0.222

MTA: mineral trioxide aggregate.

Total sample size *N* = 44 (NeoPUTTY = 22; MTA = 22). Overall treatment success 42/44 (95.5%).

Values represent number of successful cases/total cases within each category [*n*/*N* (%)].

Statistical significance at *p*-value < 0.05.

## Discussion

Full coronal pulpotomy has emerged as a definitive treatment modality for managing mature permanent teeth with irreversible pulpitis. Histopathologic evidence indicates that pulpal inflammation is often localized to the coronal pulp, while the radicular pulp may remain vital and capable of healing after removal of infected tissue [4].

Several clinical and histological studies support pulpotomy as a viable treatment for irreversible pulpitis. Eghbal et al. demonstrated histological healing and dentin bridge formation after MTA pulpotomy in mature permanent molars with irreversible pulpitis [28], while Taha and Abdelkhader reported very high success rates following full pulpotomy in mature permanent molars with symptoms indicative of irreversible pulpitis, with 100% clinical and 98.4% radiographic success at 1 year [29]. A randomized trial by Galani et al. showed comparable success between MTA pulpotomy and RCT, with lower postoperative pain after pulpotomy [30]. Systematic reviews by Aguilar and Linsuwanont, Cushley et al., and Ather et al. reported success rates ranging from approximately 86% to over 90%, supporting pulpotomy as a predictable treatment for irreversible pulpitis [31–33]. Emerging evidence in primary teeth also shows favorable outcomes. A recent systematic review and meta-analysis by Chawla et al. [34] reported no significant difference between pulpotomy and pulpectomy, including studies by Sabbagh et al. (RCT) [35] and Hu et al. (retrospective cohort) [36], both demonstrating high success rates with calcium silicate–based materials.

Consistent with these findings, the present randomized clinical study demonstrated a high 12-month clinical and radiographic success rate of 95.5% for pulpotomy in mature permanent molars. Postoperative pain reduced markedly within 24 h, reflecting rapid resolution of pulpal inflammation. Also, healing of baseline periapical radiolucencies was observed, supporting the capacity of the radicular pulp to recover following removal of inflamed coronal tissue. Similar radiographic resolution after pulpotomy has been reported by Qudeimat et al. and Taha et al. in mature permanent teeth with irreversible pulpitis [27, 29]. It should be emphasized, however, that the absence of statistically significant differences between the two materials should not be interpreted as evidence of equivalence. With only two total failures recorded across 44 teeth, the present study was not adequately powered to draw definitive comparative conclusions, and the findings should be regarded as preliminary and exploratory rather than confirmatory.

The success of pulpotomy is strongly influenced by the biological properties of the pulp-capping material. MTA has long been considered the reference standard. However, drawbacks such as difficult handling, prolonged setting time, and potential discoloration have encouraged the development of newer premixed calcium silicate biomaterials such as NeoPUTTY. Its premixed syringe formulation enables ready-to-use delivery with improved handling characteristics and reduced chair-side time, which may be particularly beneficial in children. The material sets in the presence of moisture from dentinal tubules and pulpal tissues and exhibits immediate wash-out resistance, allowing the final restoration to be placed immediately after material placement without risk of early material disintegration. NeoPUTTY also demonstrates one of the highest radiopacity values among calcium silicate–based materials (8.1 mm Al), exceeding ISO requirements and facilitating clear radiographic identification of the material [13, 14]. In vitro studies have further shown that NeoPUTTY can penetrate dentinal tubules to a greater extent than some other calcium silicate materials, which may enhance interfacial adaptation and sealing ability [37].

Recent clinical studies evaluating NeoPUTTY as a pulpotomy medicament have demonstrated favorable outcomes, particularly in primary teeth. Alqahtani et al. conducted a randomized clinical trial comparing NeoPUTTY with NeoMTA-2 in primary molar pulpotomies and reported clinical and radiographic success rates of 97.1 and 92.8%, respectively, with no significant difference between materials [13]. Similarly, Acharya et al. compared NeoPUTTY with formocresol in a randomized clinical trial in primary molars and reported clinical and radiographic success rates of 96 and 92%, indicating superior clinical performance of the bioceramic material [14]. In another randomized clinical trial, Talekar et al. compared NeoPUTTY with NeoMTA Plus and Biodentine for pulpotomy in primary molars and reported success rates exceeding 90% over a 24-month follow-up, with no statistically significant difference among the calcium silicate–based materials [15]. Evidence in mature permanent teeth is more limited but promising. A randomized clinical trial by Taha et al. evaluated NeoPUTTY in mature permanent molars with symptomatic irreversible pulpitis and reported a success rate of 98.9% for full pulpotomy and 84.5% for partial pulpotomy at 12 months [19].

In agreement with these preliminary findings, the present study detected no statistically significant difference between NeoPUTTY and MTA in clinical and radiographic outcomes. This indicates that appropriate case selection, effective hemostasis, and an adequate coronal seal are more critical determinants of success than the specific calcium silicate material used; however, this remains a hypothesis to be confirmed in larger studies. NeoPUTTY demonstrated practical handling advantages, but a structured objective assessment of handling was not performed.

Several clinical factors were evaluated for their influence on treatment outcomes. Jaw location (maxillary vs. mandibular), sex, duration of preoperative pain, hemostasis time, caries depth, and Wolters’ grade of pulpitis severity were not significantly associated with treatment success in this small sample. The choice of 5% NaOCl for hemostasis in the present study is consistent with its prior use in pulpotomy protocols for permanent molars with irreversible pulpitis [27], and falls within the 0.5–5% concentration range recommended by the ESE position statement for vital pulp therapy. The concentration of NaOCl used has been considered not to be a concern for the vitality of the exposed radicular pulp, while providing simultaneous hemostatic and disinfectant effects at the pulp wound interface [26]. With respect to hemostasis, the maximum hemostasis time recorded was 15 min. High success rates were observed across all hemostasis time categories, supporting observations by Bogen et al. and Linsuwanont et al. that hemostasis duration alone should not contraindicate pulpotomy once hemostasis is achieved [38, 39]. Similarly, the favorable outcomes observed across different caries depths and Wolters’ grades of pulpitis severity in the present study are consistent with findings reported by Aldeen et al. [40] and Jiménez-Martín et al. [41], respectively, suggesting that clinical signs and symptoms may not accurately reflect the histological status or true extent of pulpal inflammation.

The strengths of the present study include its randomized design, standardized treatment protocol, comparable baseline characteristics, and systematic evaluation of multiple clinical parameters, including hemostasis time, caries depth, pulp status, and postoperative pain. However, certain limitations should be acknowledged. First, the sample size was relatively modest, owing to the strict inclusion criteria and the limited number of eligible cases during the study period. Second, follow-up was limited to 12 months. Third, histologic confirmation of pulpal healing was not feasible. Fourth, handling characteristics were not formally assessed; therefore, the perceived convenience of NeoPUTTY’s premixed formulation represents an operator-based clinical impression rather than a measured outcome. Fifth, with only two treatment failures observed, analyses of prognostic clinical variables were inherently underpowered and should be considered descriptive rather than confirmatory. Sixth, the study was not prospectively registered prior to participant enrollment, which is a recognized methodological limitation. To ensure transparency, the study was subsequently registered, no protocol amendments were made after data collection, and all prespecified outcomes, methods, and results are reported in full without modification.

Future research should include larger, adequately powered multicenter randomized clinical trials with extended follow-up to confirm the long-term outcomes of NeoPUTTY pulpotomy in mature permanent teeth. Trials designed as non-inferiority or equivalence studies are required before definitive conclusions can be drawn regarding its comparability with MTA. Future studies should use validated tools, such as operator satisfaction scales and placement-time measurements, to compare the handling properties of NeoPUTTY quantitatively. Furthermore, investigations exploring pulpal biomarkers as predictors of treatment outcome and healing potential may help refine case selection and optimize vital pulp therapy protocols in mature permanent teeth.

## Conclusion

Within the limitations of this randomized clinical study and 12-month follow-up, no statistically significant differences were detected between NeoPUTTY and MTA in postoperative pain reduction or clinical/radiographic success after full pulpotomy in mature permanent molars with irreversible pulpitis in children aged 9–14 years. In this sample, both materials demonstrated a high overall success rate of 95.5% at 12 months. Although NeoPUTTY’s premixed formulation was perceived by the operator as clinically convenient, handling characteristics were not formally measured and should therefore be interpreted as clinical impressions rather than measured outcomes. Larger, adequately powered non-inferiority or equivalence trials with longer follow-up are required before NeoPUTTY can be confirmed as a clinically equivalent alternative to MTA for this indication.

## Data Availability

The data supporting the findings of this study are available from the corresponding author upon reasonable request.
